# Calcium electroporation for treatment of sarcoma in preclinical studies

**DOI:** 10.18632/oncotarget.24352

**Published:** 2018-01-30

**Authors:** Anna Szewczyk, Julie Gehl, Malgorzata Daczewska, Jolanta Saczko, Stine Krog Frandsen, Julita Kulbacka

**Affiliations:** ^1^ Department of Animal Developmental Biology, Institute of Experimental Biology, University of Wroclaw, Wroclaw, Poland; ^2^ Center for Experimental Drug and Gene Electrotransfer (C*EDGE), Department of Clinical Oncology and Palliative Care, Zealand University Hospital, Roskilde, Denmark; ^3^ Department of Clinical Medicine, Faculty of Health and Medical Sciences, University of Copenhagen, Copenhagen, Denmark; ^4^ Department of Oncology, Herlev and Gentofte Hospital, University of Copenhagen, Herlev, Denmark; ^5^ Department of Medical Biochemistry, Wroclaw Medical University, Wroclaw, Poland

**Keywords:** calcium electroporation, sarcoma, PMCA, calcium channels, cytoskeleton

## Abstract

Calcium electroporation (CaEP) describes the use of electric pulses (electroporation) to transiently permeabilize cells to allow supraphysiological doses of calcium to enter the cytosol. Calcium electroporation has successfully been investigated for treatment of cutaneous metastases in a clinical study. This preclinical study explores the possible use of calcium electroporation for treatment of sarcoma.

A normal murine muscle cell line (C2C12), and a human rhabdomyosarcoma cell line (RD) were used in the undifferentiated and differentiated state. Electroporation was performed using 8 pulses of 100 μs at 600–1000 V/cm; with calcium (0, 0.5, 1, and 5 mM). Viability was examined by MTS assay, intracellular calcium levels were measured, and expression of plasma membrane calcium ATPase (PMCA) was investigated using western blotting. Calcium/sodium exchanger (NCX1), ryanodine receptor (RyR1) expression and cytoskeleton structure (zyxin/actin) were assessed by immunofluorescence. CaEP efficiency on RD tumors was tested *in vivo* in immuno-deficient mice.

CaEP was significantly more efficient in RD than in normal cells. Intracellular Ca^2+^ levels after CaEP increased significantly in RD, whereas a lower increase was seen in normal cells. CaEP caused decreased expression of PMCA and NCX1 in malignant cells and RyR1 in both cell lines whereas normal cells exhibited increased expression of NCX1 after CaEP. Calcium electroporation also affected cytoskeleton structure in malignant cells.

This study showed that calcium electroporation is tolerated significantly better in normal muscle cells than sarcoma cells and as an inexpensive and simple cancer treatment this could potentially be used in connection with sarcoma surgery for local treatment.

## INTRODUCTION

Rhabdomyosarcoma is the malignant tumor derived from myoblasts and mainly affects children and young adults. Due to the most frequent localization in muscle tissue, tumor resection often impacts functional outcomes [1, 2]. The function of muscle fibers is based on electrical stimulation and calcium signaling [3]. Calcium plays a crucial role in physiological processes such as cell cycle, proliferation, differentiation, apoptosis, and necrosis [4]. Normally, the concentration of calcium ions [Ca^2+^] in the cytoplasm is maintained in the range of 10–100 nM, while the extracellular Ca^2+^ concentration is 1–2 mM. Calcium metabolism is tightly regulated by numerous pumps and ion channels located in the cell membrane, including PMCA (Plasma membrane calcium ATPase), SERCA (Ca^2+^ pump of sarcoplasmic reticulum), NCX (Na^+^/Ca^2+^ exchanger), and RyR (ryanodine receptor) [5]. Drastic increases in intracellular calcium ion concentration may affect cytoskeleton structure and mitochondria function, which cause cell swelling and in consequence lead to cell death [6].

Electroporation (EP), i.e. short high-voltage electric pulses causing transient cell membrane permeabilization, can be used to change intracellular calcium ions concentration (calcium electroporation, CaEP [7]) and may affect normal and malignant cells differently. Previous studies have shown that malignant cells are more sensitive to calcium electroporation than normal cells *in vitro* and *in vivo* [8–10]. It has also been shown that calcium electroporation is associated with acute and severe ATP depletion across tested cell lines (H69 – human small-cell lung cancer, SW780 - human bladder cancer, HT29, Human colon cancer, MDA-MB231 – human breast cancer, U937 – human leukemia, DC-3F - transformed Chinese hamster lung fibroblast cell line as well as HDF-n - primary normal human dermal fibroblasts) [7, 11–13]. Interestingly, pretreatment ATP levels did not vary significantly between cell lines indicating that it may not be the pretreatment ATP level but rather sensitivity to depletion which determines impact on viability. In support of this, in a study on 3D spheroids, we observed ATP depletion in both a normal and malignant cell spheroids. However, whereas viability in normal cell spheroids was unperturbed after calcium electroporation, malignant cell spheroids were severely affected [12].

We hypothesize that different composition of the cell membrane and cytoskeleton structure, as well as dissimilar ion channel expression might reveal various reactions between normal and malignant cells after calcium electroporation. Indeed, the differential response to calcium electroporation could also be associated with cell differentiation.

In this study, we investigated the effect of calcium electroporation on normal and malignant muscle cells, undifferentiated and differentiated as well as under different experimental conditions (suspended and attached cells). We also investigated if a difference in treatment response between normal and malignant cells was correlated to differential expression of ion channel proteins and changes of cell structures. Finally, we studied the influence of calcium electroporation on rhabdomyosarcoma tumors *in vivo*.

## RESULTS

We have previously shown that calcium electroporation (CaEP) significantly decreased sarcoma cells viability and led to apoptotic cell death. Interestingly, we also demonstrated higher survival ratio in normal muscle cells after treatment compared with sarcoma cells [8]. In this study, we have confirmed these results by showing higher toxicity of calcium electroporation on sarcoma cells (RD) compared to normal muscle cells (C2C12). Additionally, and not previously shown, differentiation process increased cell survival ratio for both cell lines. Furthermore, we have compared the expression of plasma membrane calcium ATPase (PMCA), sodium-calcium exchanger (NCX), and ryanodine receptor (RyR) as well as structural changes in the two cell lines before and after treatment, which is all described below.

### Influence of CaEP *in vitro*

**Figure 1:**
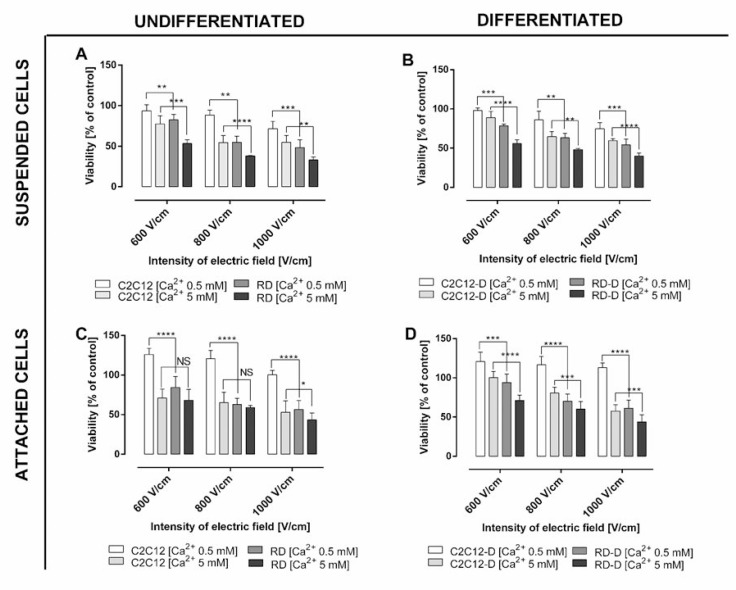
The viability assay of normal and malignant cells in respectively undifferentiated and differentiated state after electroporation with/without calcium ions (**A**) Undifferentiated normal mouse myoblast (C2C12) and malignant human rhabdomyosarcoma (RD) treated in suspension; (**B**) differentiated C2C12-D and RD-D cells treated in suspension; (**C**) differentiated, adherent normal mouse myoblast (C2C12) and malignant human rhabdomyosarcoma (RD); (**D**) differentiated, adherent C2C12-D and RD-D after treatment with calcium ions (0.5 mM and 5 mM) and electroporation (600, 800, and 1000 V/cm, respectively). Viability was determined using MTS assay 1 day after treatment. Results are presented as the percentage of control cells (non-electroporated cells without calcium ions addition). Mean ± SD, *n* ≤ 6; ^*^p < 0.05, ^**^p < 0.01, ^***^p < 0.001, ^****^p < 0.0001, NS-not significant.

We investigated the effect of calcium electroporation *in vitro* in normal muscle cells (C2C12) and sarcoma cells (RD), respectively undifferentiated and differentiated, as well as in suspension and attached (Figure 1). Three electroporation parameters (600 V/cm, 800 V/cm and 1000 V/cm) were tested in the presence of 0.5 mM and 5 mM calcium. As expected, calcium electroporation induces cell death, and the highest electric field combined with the highest calcium concentration tested caused the lowest cell survival for both cell lines (*p* < 0.01). The viability of RD sarcoma cells decreased after calcium electroporation in all the investigated cases. Interestingly, the normal C2C12 cells were significantly less affected than the RD cells (*p* < 0.05), except in two treatment combinations (undifferentiated, attached cells treated with 5 mM calcium electroporation using 600–800 V/cm; Figure 1C).

The difference in effect of calcium electroporation between undifferentiated and differentiated cells has not previously been compared. In this study we showed that differentiated cells (both cell lines) had 5–10% higher survival ratio than undifferentiated cells; however, not significantly different (Figure 1B and 1D). After differentiation, the C2C12 cells (C2C12-D) still indicated better tolerance to calcium electroporation than RD cells after differentiation (RD-D) (*p* < 0.01).

When comparing the effect of calcium electroporation on attached and suspended cells, it seemed that attached cells tolerated the treatment better than suspended cells (around 20% higher survival of normal cells and 10% higher survival of malignant cells); however not significantly different. Interestingly, attached C2C12 and C2C12-D have 25% higher viability after 0.5 mM calcium electroporation (all EP parameters) compared to untreated cells (*p* < 0.001; Figure 1C and 1D). This is not seen with malignant cells, why the largest difference between normal and malignant cells is seen for attached cells treated with 0.5 mM calcium electroporation (*p* < 0.001).

### Intracellular calcium level after calcium electroporation

Since calcium electroporation was less effective in normal than malignant muscle cells, we investigated intracellular calcium level in both cell lines, undifferentiated (C2C12, RD) and differentiated (C2C12-D, RD-D), before and after calcium electroporation.

**Figure 2:**
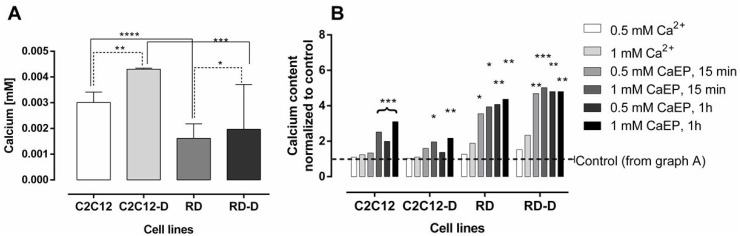
The intracellular calcium level after electroporation with Ca^2+^ of normal and malignant cell lines The assessment of intracellular calcium levels in (**A**) untreated undifferentiated normal mouse myoblast – C2C12; differentiated normal mouse myoblast – C2C12-D; undifferentiated human rhabdomyosarcoma – RD; differentiated human rhabdomyosarcoma – RD-D and (**B**) after treatment with calcium (0.5 mM and 1 mM) with or without electroporation (EP; 1000 V/cm) measured 15 min and 1 h after treatment. Results are shown where control is normalized to 1 for each cell line. Mean ± SD, *n* ≤ 5. Results statistically significant compared to control cells, ^*^p < 0.05, ^**^p < 0.01, ^***^p < 0.001, ^****^p < 0.0001.

Figure 2A shows the intracellular calcium content in untreated normal and malignant cell lines, both differentiated and undifferentiated. Calcium content was approximately twice as high in untreated C2C12 as in RD cells (*p* < 0.0001) and in C2C12-D as in RD-D (*p* < 0.001). The differentiation process also increased calcium content in both cell lines, especially in C2C12-D where the calcium level was 43% higher than in C2C12 (*p* < 0.01) and 22% higher in RD-D than in RD (*p* < 0.05).

After calcium electroporation (15 min and 1 hour after), we observed a significantly higher calcium concentration in RD and RD-D cells (*p* < 0.5; Figure 2B). There was observed even 4-fold increased calcium level. In the normal C2C12 and C2C12-D cells, intracellular calcium concentration also increased after calcium electroporation, but less than in the RD and RD-D cells, and not significantly for all tested parameters compared with untreated controls (see Figure 2B). In the differentiated C2C12-D cells only the highest calcium concentration (1 mM) with electroporation caused significantly higher intracellular calcium concentration compared with untreated control (*p* < 0.05). Treatment with calcium alone did not significantly change the intracellular calcium level in any of the cell lines.

The difference in intracellular calcium after calcium electroporation in normal and malignant cells could indicate that there, amongst other, is a difference in how the cells remove the high calcium concentrations to re-establish the calcium homeostasis. We therefore investigated some of the calcium pumps and channels in the cells.

### Expression of PMCA proteins

**Figure 3:**
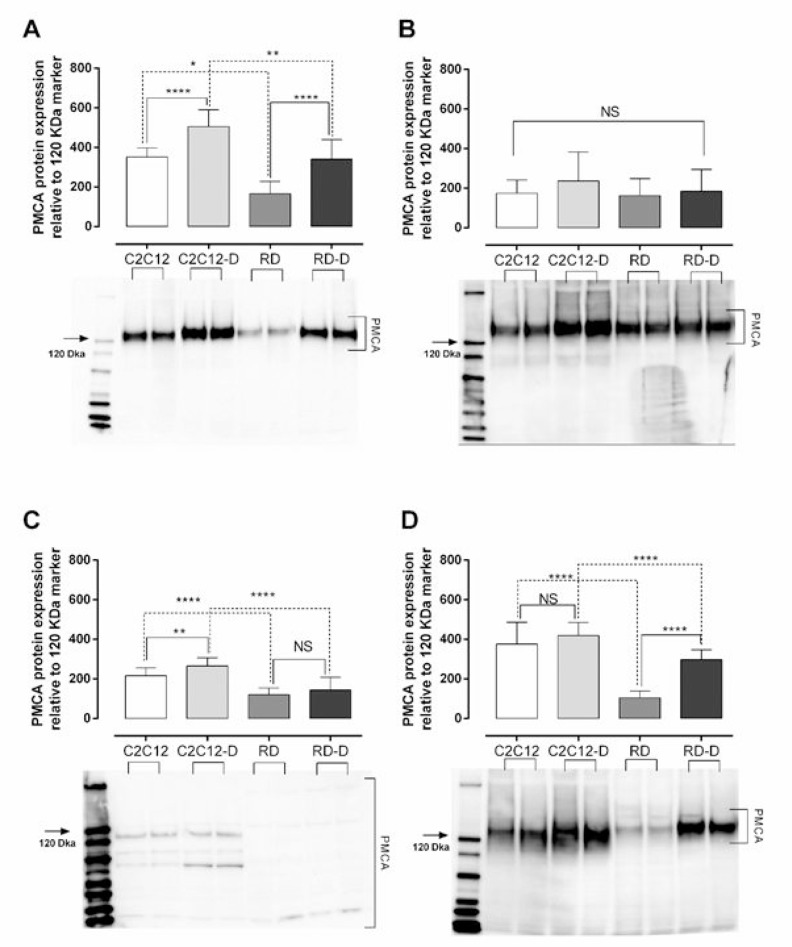
Plasma membrane calcium ATPase (PMCA) protein expression in normal muscle (C2C12) and malignant rhabdomyosarcoma (RD) cells (**A**) Total PMCA, (**B**) PMCA isoform 1, (**C**) PMCA isoform 3, (**D**) and PMCA isoform 4 protein expression in undifferentiated normal mouse myoblast – C2C12; differentiated normal mouse myoblast – C2C12-D; undifferentiated human rhabdomyosarcoma – RD; differentiated human rhabdomyosarcoma – RD-D measured by western blotting. Representative blot with each sample loaded twice is shown below the graph. The protein level measured is relative to the 120 kDa maker (marked with an arrow). Mean ± SD, *n* = 3 each investigated in duplicates. ^*^p < 0.05, ^**^p < 0.01, ^***^p < 0.001, ^****^p < 0.0001, NS – not significant.

Plasma membrane calcium ATPase (PMCA) is the main pump responsible for removal of calcium ions out of the cell and thereby maintain proper intracellular calcium concentration [14]. We have investigated the expression of three of four isoforms of PMCA protein (PMCA1, PMCA3 and PMCA4) as well as the expression of all PMCA isoforms together (PMCAs) in normal (C2C12, C2C12-D) and malignant (RD, RD-D) cells. PMCA1 and PMCA4 are ubiquitously expressed and described as “housekeeping” isoforms [15] while PMCA 3 protein is predominantly expressed in brain and skeletal muscle [16] and is more efficient in extruding calcium from the cells [17]. The PMCA 2 was omitted since it mostly expressed in the nervous system and mammary gland [18]. The total PMCA1-4 protein expression (Figure 3A) was significantly higher in untreated normal cells than in malignant cells: twice as high in C2C12 than RD (*p* < 0.05), and 48% higher in C2C12-D than RD-D (*p* < 0.01). Interestingly, the differentiation process increased PMCA1-4 expression by 44% for C2C12-D (*p* < 0.0001) and even doubled for RD-D (*p* < 0.0001). When estimating the PMCA isoforms individually, the normal cells presented higher expression of all the examined isoforms (PMCA1, PMCA3, PMCA4) compared to malignant cells, however not significantly for PMCA1 (Figure 3B). PMCA3 expression was almost twice as high in C2C12 and C2C12-D compared with RD and RD-D (180–185%, *p* < 0.0001). The expression of PMCA4 (Figure 3D) was 3.6-fold higher in C2C12 compared with RD (*p* < 0.0001) and 40% higher in C2C12-D than RD-D (*p* < 0.0001).

### Expression of NCX and RyR proteins

**Figure 4:**
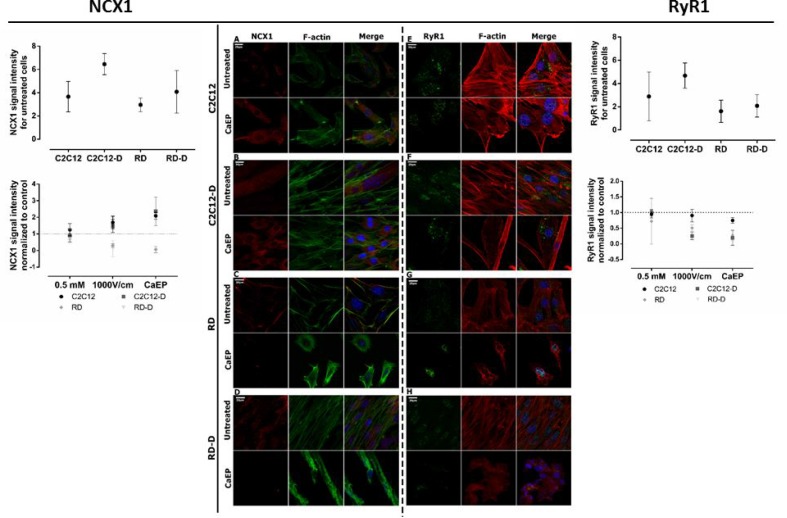
Immunofluorescent visualization of calcium/sodium exchanger (NCX1) and ryanodine receptor (Ryr1) in normal (C2C12, C2C12-D) and malignant (RD, RD-D) cells after exposition to CaEP protocol (electric field intensity: 1000 V/cm and 0.5 mM Ca^2+^) The left panel: CLSM images present changes in NCX1 (red) intracellular localization and signal intensity between untreated and CaEP cell lines: C2C12 (**A**), C2C12-D (**B**), RD (**C**), RD-D (**D**). The graph shows signal intensity for untreated cell lines (above) and 3 therapy conditions (below): Ca^2+^ incubation, EP only and CaEP normalized to untreated cells. The right panel: confocal images present changes in RyR1 (green) signal intensity between untreated and CaEP cell lines: C2C12 (**E**), C2C12-D (**F**), RD (**G**), RD-D (**H**). Both NCX1 and RyR1 protein colocalized with F-actin and cellular nucleus. The graphs NCX1 (left) and RyR1 (right) show a signal intensity for untreated cell lines (above) and 3 therapy conditions (below): Ca^2+^ incubation (0.5 mM), EP only (1000 V/cm) and CaEP (0.5 mM; 1000 V/cm) normalized to untreated cells. Fluorescent signal was detected 24 h after CaEP application; 20 μm; *n* = 3–5. The signal intensity was analyzed by ImageJ software.

The protein expression of two calcium channels, the sodium-calcium exchanger (NCX1) and the ryanodine receptor (RyR1) were also analyzed. NCX is a sodium-calcium exchanger located in the plasma membrane that extrude Ca^2+^. In this study, NCX1 proteins were observed in the cytoplasm excluding nucleus. In the expression of NCX1, no significant difference was seen between untreated C2C12 and RD, but a higher expression in C2C12-D than RD-D, *p* < 0.05 (Figure 4A–4D). After calcium electroporation, NCX1 expression increased in normal C2C12 (*p* < 0.01) and C2C12-D (*p* < 0.001) cells. NCX1 expression also increased after treatment with electroporation alone in C2C12-D cells (*p* < 0.01) (Supplementary Figure 1B). Interestingly, unlike in the normal cells, the NCX1 protein expression significantly decreased in RD and RD-D cells after calcium electroporation (*p* < 0.001, *p* < 0.001) (Figure 4C and 4D)as well as after electroporation alone (*p* < 0.01, *p* < 0.001) (Supplementary Figure 1C and 1D). The lowered NCX1 expression and thereby lowered contribution in calcium removal in RD and RD-D cells after calcium electroporation may explain part of the problem of proper calcium homeostasis maintaining after treatment, which leads to cell death.

Ryanodine receptor is responsible for calcium-induced calcium release from calcium stores in the sarcoplasmic reticulum (SR). In the normal C2C12 cells, RyR1 expression was at the same level in untreated control as in cells treated with calcium alone, electroporation alone, and calcium electroporation (Figure 4E and Supplementary Figure 1E). However, in the differentiated C2C12-D cells, where the expression of RyR1 in untreated cells was higher than in C2C12 cells (*p* < 0.01), a decreased RyR1 expression was seen after CaEP (*p* < 0.05) (Figure 4F and Supplementary Figure 1F). Untreated malignant (RD and RD-D, Figure 4G–4H) cells showed lower RyR1 expression than normal cells (C2C12 and C2C12-D, *p* < 0.05). There was no significant difference between RyR1 signal in RD and RD-D as between C2C12 and C2C12-D cells; however, there was still a lower RyR1 expression after CaEP treatment in the RD and RD-D cells (*p* < 0.05). Unlike NCX1 expression, mostly intranuclear expression of RyR1 was observed after treatment with electroporation and CaEP (Supplementary Figure 1G and 1H; Figure 4G and 4H).

### Cytoskeleton structure after calcium electroporation

**Figure 5:**
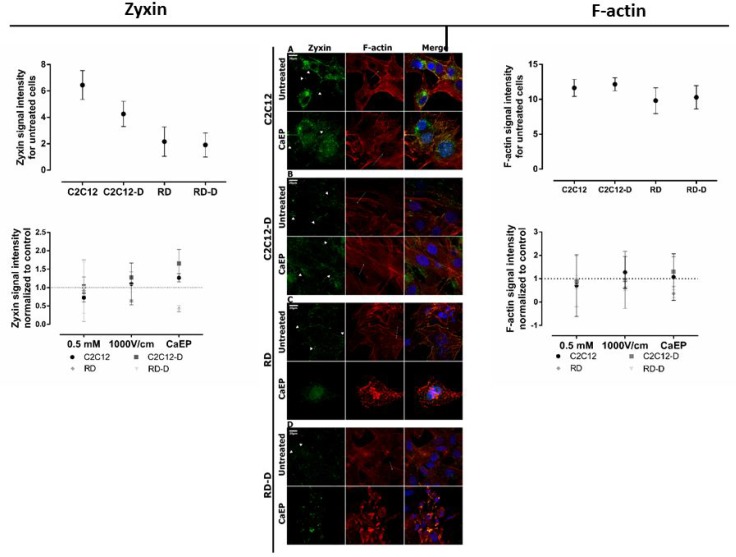
CLSM visualization of the rearrangement of zyxin and F-actin structure in normal (C2C12, C2C12) and malignant (RD, RD-D) cells Middle panel presents immunofluorescence evaluation of zyxin (green) and actin (red) fibers expression for undifferentiated cell lines: C2C12 (**A**), RD (**C**) and differentiated cell lines: C2C12-D (**B**), RD-D (**D**) 24 h after calcium electroporation application. The white short arrows indicate focal adhesions, white long arrows indicate actin stress fibers; 20 μm; *n* = 3–5. The graphs Zyxin (left) and F-actin (right) show zyxin signal intensity and actin signal intensity for untreated cell lines (above) and 3 therapy conditions (below): Ca^2+^ incubation (0.5 mM), EP only (1000 V/cm) and CaEP (0.5 mM; 1000 V/cm) normalized to untreated cells. The signal intensity was analysed by ImageJ software.

We also investigated cell morphology after treatment. First, F-actin and zyxin staining were performed to examine cytoskeleton before and after calcium electroporation (Figure 5 and Supplementary Figure 2).

Differences in cytoskeleton structure between normal and malignant cells was seen. F-actin filaments in C2C12 and C2C12-D cells were well-organized in higher-order structure, forming a stable net. The zyxin protein were co-localized with actin fibers on the membrane edges (Figure 5A–5B). It is evident that calcium electroporation stimulated zyxin expression in focal adhesion as well as actin stress fibers tension in normal cells, which is also seen in the graph where zyxin signal intensity increased after calcium electroporation (*p* < 0.05). RD and RD-D cells exhibited inferior organized F-actin fibers assembly with looser meshwork than presented in normal cells (Figure 5C–5D). The images also show that malignant cells (untreated and after incubation with 0.5 mM calcium) were rich in focal adhesion and lamellipodium which is responsible for cancer cells migration (Figure 5C and 5D; Supplementary Figure 2C and 2D). Both actin and zyxin were expressed strongly in the membrane protrusions. Electroporation with and without calcium disassembled actin cytoskeleton and inhibited zyxin expression. As seen in the graph, the intensity of the zyxin signal decreased (*p* < 0.05) after treatment with calcium electroporation, opposite the normal cells. RD and RD-D cells were small and shrunk (Figure 5C–5D and Supplementary Figure 2C and 2D).

### Ultrastructure analysis by TEM method

**Figure 6:**
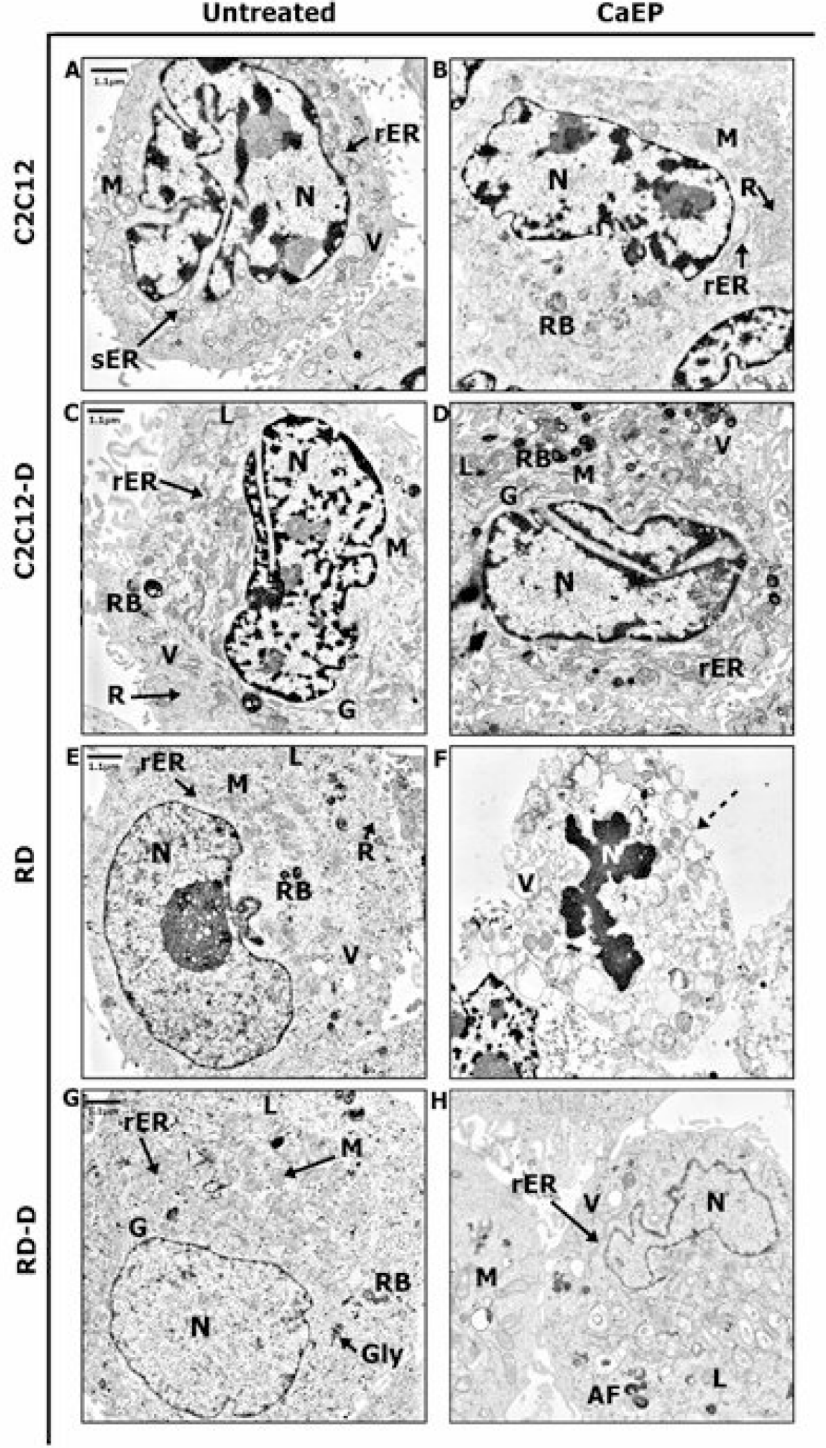
TEM figures presenting changes of the ultrastructure in normal (C2C12, C2C12-D) and malignant (RD, RD-D) rat muscle cells The representative images present structure of intracellular organelles of untreated cells: C2C12 (**A**), C2C12-D (**C**), RD (**E**), RD-D (**G**) and 24 h after CaEP (1000 V/cm, 8 pulses, 100 μs): C2C12 (**B**), C2C12-D (**D**), RD (**F**), RD-D (**H**). Figure shows the results of one representative out of three independent experiments. N-nucleus; M-mitochondrion; G-Golgi apparatus; V-vacuoles; sER-smooth endoplasmic reticulum; rER-rough endoplasmic reticulum; R-free ribosomes; L-lysosomes; RB-residual bodies; dotted arrow indicates cell membrane disruption.

Ultrastructure of normal and malignant cells was subjected to precise assessment by transmission electron microscope (TEM). Untreated normal C2C12 cells revealed the model composition of muscle cell structure with large nucleus with visible heterochromatin and active nucleolus, well-developed smooth endoplasmic reticulum (sER), and numerous mitochondria and lysosomes (Figure 6A). CaEP did not affect the cell structure but the sER was replaced by rough endoplasmic reticulum (rER) and the number of free ribosomes increased drastically (Figure 6B). Differentiated normal cells (C2C12-D) exhibited increased amount of rough endoplasmic reticulum, free ribosomes, and Golgi apparatus (Figure 6C). The nucleus was abounding in heterochromatin which might be categorized as facultative heterochromatin containing genes that are silent upon differentiation process [19]. Untreated malignant cells (RD, RD-D) were rich in mitochondria and ribosomes, which is a distinguishing mark of cancer cells and promotes their survivability (Figure 6E and 6G) [20]. After calcium electroporation of C2C12-D cells, an increased variety of secretory vesicles such as vacuoles, lysosomes, and residual bodies was seen (Figure 6D). These vesicles were also observed in malignant differentiated cells (RD-D) after CaEP as well as folded nucleus. Additionally, large, swollen mitochondria appeared with abnormal crista and distended rER (Figure 6H). In RD cells, a significant apoptotic reaction was observed after calcium electroporation (Figure 6F) where the cell membrane integrity was strongly disrupted. Moreover, plenty of vacuoles and folded nuclei were also observed in these cells and particular cell organelles were difficult to identify. It all indicated the cell death processes confirmed by MTS assay (Figure 1).

### Influence of calcium electroporation *in vivo*

**Figure 7:**
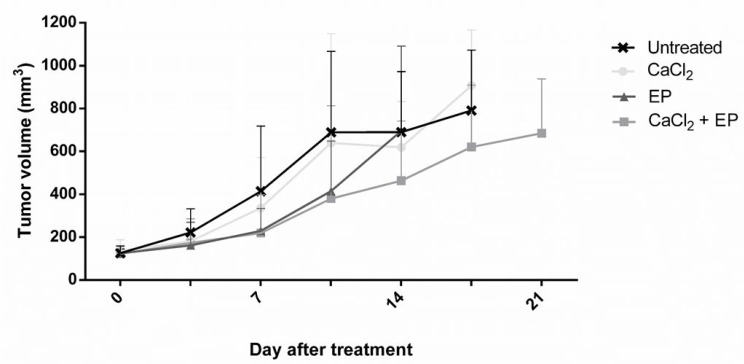
Electroporation with calcium ions of rhabdomyosarcoma tumor *in vivo* Human rhabdomyosarcoma tumors on nude mice were treated with calcium, calcium electroporation, electroporation alone, untreated. Mice were randomized and treated when tumors reached a volume of 85 mm^3^. Tumor volume was measured 2 times per week. Mean ± SD, *n* = 10 before treatment, *n* ≥ 6 is shown in the graph.

Due to the promising *in vitro* results of calcium electroporation, the effect of the treatment was also tested *in vivo*. RD tumors, grown subcutaneously on NMRI-*Foxn1^nu^* mice, were treated with calcium injection and electroporation using 1000 V/cm. Controls were treated with CaCl_2_ injection alone, electroporation alone, and untreated (Figure 7). The largest difference in tumor volume between the untreated and CaEP treated was observed 10 days after treatment (45% decrease of tumor volume); however, tumors treated with calcium electroporation was not significantly different in volume from any of the control groups. The decrease of tumor volume after calcium alone and electroporation alone was unnoticeable.

## DISCUSSION

In this study, we have investigated the effect of calcium electroporation on normal and malignant muscle cells *in vitro* and *in vivo*. This study has confirmed our previous work about cytotoxic effect of calcium electroporation on malignant muscle cells, which increased with increasing calcium concentration and/or applied voltage, as well as less effect was shown in normal muscle cells [8]. Moreover, this study is the first report about difference in response to CaEP between non-differentiated and differentiated cells. Normal muscle cells, both undifferentiated and differentiated, preserved significantly higher cell viability than undifferentiated and differentiated malignant cells, respectively. Interestingly, there seems to be a slightly higher viability of differentiated cells than undifferentiated cells after treatment (significant only for parameters: 800V/cm with 5 mM Ca^2+^; *p* < 0.01) which indicate that both differentiated and undifferentiated cells should be tested for further experiments. The attached cells might also be less sensitive for CaEP than suspended cells, but it was not statistically significant.

We showed an increased intracellular calcium concentration in all cells after calcium electroporation. A drastic increase of calcium level has a profound impact on cellular components such as cytoskeleton, cell membrane, opening of mitochondrion permeability transition pores (PTP), and elevating concentration of reactive oxygen species (ROS) [9]. Interestingly, we only confirmed significantly higher calcium content in RD and RD-D cells after CaEP using 0.5 mM calcium, not in C2C12 and C2C12-D cells. We hypothesized that this could have a background in the cell membrane structure and/or in quantity, type, and activity of ion channels and pumps [18, 21, 22]. Considering the physiological function of normal muscle cells (contraction and relaxation), they have a very efficient mechanism to maintain calcium homeostasis [23]. The results of this study indicate that plasma membrane calcium ATPase is higher expressed in normal muscle cells than malignant cells. Numerous studies have shown, that up- or down-regulation of PMCA expression is depended on cancer cells types [24–26]. Our results describe a correlation between viability after treatment, intracellular calcium content, and PMCA expression, what confirms different response to calcium electroporation in normal and malignant cells, as previously shown in other cell types [10]. The higher expression of PMCA in normal cells indicates fast and efficient removal of calcium ions from the cytosol, which could increase viability. Lower expression of PMCA in malignant cells has also previously been shown [27, 28]. However, further experiments are needed to show the PMCA expression and activity after CaEP.

We also proved that the expression of the sodium-calcium exchanger (NCX1) is lower in malignant RD/RD-D cells than normal C2C12/C2C12-D cells after calcium electroporation (*p* < 0.01). This is likely also leading to increased cytosolic Ca^2+^ and thereby lower cell viability in the malignant cells. Previous research suggested calcium removing as the main function of NCX in stimulated muscle cells [29]. In addition, NCX supports malignant cell proliferation and migration [30, 31], thus the reducing effect of CaEP on NCX expression may contribute to unveiling a new approach to fibrosarcoma treatment.

Interestingly, NCX exchange Ca^2+^ for Na^+^, and the direction of the flux depends on the electrochemical gradient of both ions [32]. Thus, a thorough analysis of the impact of calcium electroporation on the NCX flux and thereby the Na^+^ and Ca^2+^ homeostasis is highly recommended.

Muscle cells also maintain calcium homeostasis by storing calcium ions in sarcoplasmic calcium storages. The expression of ryanodine receptor (RyR), which play a crucial role in intracellular Ca^2+^ release from SR was therefore investigated. We showed a decrease of RyR1 expression in normal differentiated cells C2C12-D (*p* < 0.01) after calcium electroporation but not in RD-D cells. A reduction of RyR1 expression has previously been shown to induce cell death [33, 34], but this is not the case in this study, maybe due to the increased expression of PMCA and NCX in the normal cells. To further understand the impact of CaEP on calcium homeostasis, we suggest investigation of RyR activity, expression and activity of SERCA, and SR storage capacity should be performed.

It has previously been shown that calcium electroporation causes acute and severe ATP depletion [7, 11–13]. This study does not encompass ATP examination however we previously showed that ATP uniformly and severely depleted across cell lines as a result of calcium electroporation but normal cells seem more resilient to this depletion than malignant cells. In this study, we found clear differences in survival after calcium electroporation *in vitro* between normal and malignant cell lines which is the important outcome.

In this study, we have also investigated CaEP impact on cytoskeleton, particularly on zyxin and actin expression. Spatial structure of actin cytoskeleton is crucial for numerous processes such as cell migration, membrane extension, and extracellular signal transduction [35]. Zyxin is the main protein regulating actin cytoskeleton and stimulating spatially restricted actin polymerization [36, 37], which is strongly expressed in fast-growing ends of actin filaments e.g. lamellipodia [38]. It exports signal between nucleus and cell adhesion sites and creates cell-cell focal adherent junctions [39]. According to previous studies, the membrane components spatial rearrangement, caused by electroporation, may affect the focal adhesion junctions and disturb zyxin expression leading to F-actin bundles unbinding [40, 41]. Our study is the very first to demonstrate that the impact of CaEP on zyxin distribution. In malignant RD/RD-D cells, this lead to loss of cell-cell contact and adherence. Interestingly, normal C2C12/C2C12-D cells exhibit high zyxin expression and fast recovery processes induced at zyxin accumulation spots [42]. The higher viability rate of adherent cells is likely due to robust cytoskeleton. In contrast to suspended cells, they create well-organized cytoskeleton structure with cell-cell junction, providing efficient cooperation and stable filaments assembly. In this unique study, we differentiated cells to investigate the importance of cell-cell contacts and development stage in calcium homeostasis after calcium electroporation treatment, which to our knowledge have not previously been shown. Our results show that there is a clear difference in the cytoskeleton structure after differentiation of normal and malignant cells, which might affect the cell response to calcium electroporation.

We also investigated changes caused by calcium electroporation using transmission electron microscope (TEM). The most apparent differences caused by CaEP were visible in the malignant RD cells, mainly in damaged cell membrane and vacuolisation of cell compartments, which suggests late apoptosis. The enlarged, swollen mitochondria with disarrangement of cristae in RD-D cells appearing after calcium electroporation may indicate abnormal energy metabolism and ATP depletion leading to cell death [43, 44], what correlates with the MTS results and previous studies which showed that ATP levels were acutely and severely depleted after electroporation alone as well as after calcium electroporation [7, 10]. Indeed, the mitochondrial function, including ATP production and involvement in the maintenance of intracellular calcium homeostasis, has previously been shown to be altered in cancer cells [45, 46]. This might also influence the effect of calcium electroporation. In contrast, CaEP strengthens metabolic activity of normal muscle cells by a number of free ribosomes that synthesize proteins essential for intracellular activity [47]. We might presume that calcium electroporation increase ribosome activity and protein expression in response to the fast upgrade of cell structures. Interestingly, after calcium electroporation, normal C2C12-D cells exhibit a variety of secretory vesicles, which may indicate cell death pathway activation [48, 49]. However, high survival ratio was confirmed by MTS assay. Thus, the cellular recycling system might play a protective role by removing damaged organelles or cytotoxic substances, such as excessive calcium ions. This mechanism combined with a large number of active ribosomes might neutralize the harmful impact of the therapy. Autophagosomes deliver cytoplasmic components (organelles, molecules) to lysosomes, where digestion processes occur. Under unfavorable conditions, autophagy is activated and promotes cell survivability [50]. Further analyses towards vesicles origin are required.

The promising effect of calcium electroporation *in vitro* was not confirmed *in vivo* when treating RD tumors on nude mice. Although, a slight decrease of the tumor volume was seen after CaEP compared with untreated control tumors, the differences were not statistically significant. The cells used was a human cell line which in *in vivo* studies cannot be injected in immunocompetent mice. The immune-incompetent mice used in this study may explain the weak tumor response. A recent study showed that calcium electroporation plays a crucial role in immune system stimulation [51–53]. This could explain the low effect of calcium electroporation *in vivo*. Another *in vivo* study proved significant tumor necrosis in 2 out of 4 tumor types treated with CaEP, which also confirms our results. Interestingly, in the same study, lower necrotic fraction was observed in normal muscle tissue directly treated with calcium electroporation compared with tumor tissue [10], which also confirms our *in vitro* data.

Electrochemotherapy has been successfully used as local treatment of a variety of cancers for a quarter-century. Yet, electrochemotherapy in rhabdomyosarcoma treatment has not been reported. Our innovative study suggests a possible novel treatment option, calcium electroporation, for sarcoma. This study clearly highlights the difference between calcium homeostasis in normal and malignant muscle cells, which is fundamental knowledge for the possible usage of this novel treatment. We have indicated that normal muscle cells more effectively re-establish calcium homeostasis, which allows them to preserve higher viability than malignant cells after calcium electroporation. Further research focused on CaEP impact on calcium maintaining system in muscle cancer as well as other muscle disorders are highly recommended. This novel anticancer therapy seems to be very promising for targeted muscle cancer treatment in near future since the equipment, agent, and procedure are easily accessible, and the low-cost of this therapy may raise standards of treatment in many countries.

## MATERIALS AND METHODS

### Cell cultivation

The *in vitro* studies utilized two cell lines: C2C12 - mouse myoblast cell line and RD - human rhabdomyosarcoma cell line, respectively undifferentiated and differentiated [54, 55]. The undifferentiated cell lines were grown in DMEM culture medium (Gibco) with 10% fetal calf serum, penicillin, streptomycin, and glucose (RD – 4.5 g/L, C2C12 – 1 g/L) at 37μ C and 5% CO_2_. Both cell lines were tested negative for mycoplasma (MycoAlert, Lonza), and the RD cell line was authenticated by short tandem repeat profiling (LGC Standards) in April 2016 showing a perfect match.

Differentiation was initiated at 70–90% cell confluence. Standard culture medium for C2C12 was changed for DMEM with 2% horse serum and 4.5 g/L glucose. For RD cells, standard culture medium was added 12-O-tetradecanoyl-phorbol-13-acetate (TPA, 100 nM in EtOH; Sigma Aldrich). Culture medium was changed daily. Cells underwent differentiation over 5 days before experiments.

### Electroporation of cells in suspension

Cells (270 μl of 6.1 × 10^6^ cells/ml) suspended in HEPES buffer (10 mM HEPES (Lonza), 250 mM sucrose, and 1 mM MgCl_2_ in sterile water) and 30 μl of CaCl_2_ (0.5, 1, or 5 mM in final concentration) or HEPES for controls incubated 5 min at 37μ C in a 4 mm cuvette (Molecular BioProducts, Inc.). Cells were exposed to 8 pulses of 99 μs, 1 Hz, and 600, 800, or 1000 V/cm using a square wave electroporator (BTX T820, Genetronics, USA). After 20 min incubation at 37μ C, cells were suspended in culture medium and seeded in 96-well plates (3.1 × 10^4^ cells per 100 μl) and incubated for 24 h at 37μ C. MTS assay was performed using Multiscan-Ascent ELISA reader (ThermoLabsystems).

### Electroporation of attached cells

In glass bottom dishes (HBSt-3512, WillCo Wells), 300 μl of cells (1.66 × 10^5^) seeded in culture medium and incubated overnight (undifferentiated cells) or 5 days (differentiated cells). Cells in HEPES buffer containing 0.5 or 5 mM CaCl_2_ were electroporated using a square wave electroporator (Cliniporator, IGEA) and a custom-made contact copper electrode device with 8 mm between the electrodes (same parameters as described above). After electroporation, plates incubated for 20 min at 37μ C before HEPES buffer was changed to 1.5 ml culture medium. Cells incubated at 37μ C for 24 h. Before MTS assay, culture medium was substituted by 100 μl medium and 20 μl MTS and incubated for 1.5 h. Cell viability was measured as described above.

### Calcium level measurement

Intracellular calcium level was measured for the two cell lines, undifferentiated and differentiated. Cells were divided into seven groups: Calcium electroporation (0.5 and 1 mM) collected 15 and 60 min after treatment, calcium alone (0.5 and 1 mM) collected after 60 min, and untreated. Cells were electroporated in suspension using 1000 V/cm. After treatment, cells were suspended in 50 μl lysis buffer for 30 min, centrifuged, and the supernatant was collected. Total intracellular calcium concentration was measured using Calcium Colorimetric Assay Kit (Bio Vision) in 96-well plates. A standard curve was generated using serial dilutions of CaCO_3_ (0–2 mg/dl). Absorbance was measured using Multiscan-Ascent ELISA reader (ThermoLabsystems) at 575 nm. The obtained results were expressed as Ca^2+^ level in mM where 1 mg/dl CaCO_3_ corresponds with 0.1 mM Ca^2+^.

### Western blot

Undifferentiated and differentiated C2C12 and RD cell lines were examined for PMCA protein expression. Cell suspensions were incubated in lysis buffer (137 mmol/L, 20 mmol/L Tris, 1 % nonidet P 40, 10% glycerol, 1 mmol/L PMSF Sigma P7626, 10 μg/ml Aprotinin Sigma, 0.5 mmol/L natrium metavanadate Sigma 590088, 1 μ/ml leupeptin Sigma L2882; pH = 8.0) on ice for 30 min with agitation. Supernatant collected after centrifugation (8,000 g × 3 min). Protein concentration were measured by BCA Protein Assay (Pierce) and 20 μg of total protein extract was mixed with Sample Load Buffer (Invitrogen) and reducing agent (Invitrogen), heated for 5 min at 90μ C, and separated on NuPAGE 3–8% Tris-Acetate Gel together with a pre-strained protein marker and a Magic marker (Novex; 150 V for 1 h). The separated protein was transferred to a PVDF membrane (Invitrogen) using wet blotting (1.5 h; 90 mA). The membrane was incubated overnight with diluent A and B (Invitrogen) in 2:3 ratio to block for unspecific binding prior to incubation (1 h) with the primary antibody. Antibodies were diluted in diluent A and B (ratio 2:1). Four primary antibodies (1:1000) were used: mouse-anti-PMCA antibody 5F10 (Thermo Fisher Scientific), mouse anti-PMCA-4 antibody JA9 (Thermo Fisher Scientific), rabbit monoclonal anti-PMCA-1 antibody EPR12029 (Abcam), and rabbit polyclonal anti-PMCA-3 antibody ab84521 (Abcam). Then, the membrane was washed in 1X Wash buffer for 2 × 10 min followed by incubation with secondary HPR conjugated antibody (1:10.000) diluted in a 2:1 mixture of diluent A and B. Unbound antibody was removed with a second wash procedure (2 × 10 min). The membrane was incubated with a Chemiluminescent Substrate Reagent Kit (Invitrogen) for 5 min with minimal light exposure. Signal was elicited using LAS-4000 Chemiluminescence and Fluorescence Imaging System (Fujitsu Life Science). Band intensity was estimated and compared to the 120 kDa band of the Magic marker (Invitrogen).

### Confocal laser microscopy study (CLSM)

Confocal microscope was used to evaluate cytoskeleton structure and calcium ion channels expression in adherent cells after calcium electroporation (0.5 mM, 1000 V/cm). Cells (10^3^/100 μl) were incubated on cover glasses in Petri dishes overnight. The treatment (calcium electroporation, calcium alone, electroporation without calcium, untreated) using Petri Pulser Electrode with 2 mm gaps between electrodes (BTX model 45–0130) was performed. Then after 24 h cells were fixed in 4% formalin, permeabilized with 0.5% Triton X-100 in PBS for 5 min, raised in PBS 3 × 5 min, blocked with 1% Bovine Serum Albumin (BSA) in PBS for 1 h. These antibodies were used: 1) to show cytoskeleton structure – primary antibody monoclonal mouse anti-zyxin (overnight incubation at 4μ C; 1:500; Abcam) and secondary antibody Fluorescein (FITC)-conjugated AffiniPure Fragment Donkey Anti-Mouse IgG (for 60 min; at room temperature; 1:100; Jackson ImmunoResearch) mixed with Alexa 546-conjugated phalloidin (at a concentration of 2 μg/ml; Life Technologies); 2) to show ryanodine receptors expression – primary antibody monoclonal rabbit anti-RyR1 (overnight at 4μ C; 1:100, Cell Signaling) and secondary antibody Cy3-conjugated AffiniPure Donkey Anti-Rabbit IgG (for 60 min at the room temperature; 1;100; Jackson ImmunoResearch) mixed with Alexa 488-conjugated phalloidin (at a concentration of 2 μg/ml; Life Technologies); 3) to show sodium/calcium exchanger expression – primary antibody monoclonal mouse anti-NCX1 (overnight at 4μ C; 1:100, Abcam) and secondary antibody Fluorescein (FITC)-conjugated AffiniPure Fragment Donkey Anti-Mouse IgG (for 60 min; at the room temperature; 1:100; Jackson ImmunoResearch) mixed with Alexa 546-conjugated phalloidin (at a concentration of 2 μg/ml; Life Technologies). DNA staining with DAPI (4,6-diamidino-2-phenylindole; 0.2 μg/ml) was performed. Cells were mounted in Fluorescence mounting medium (DAKO). For imaging, Olympus FluoView FV1000 confocal laser scanning microscope (Olympus) were used.

### Transmission electron microscopy (TEM) study

Morphological structures of cells were assessed using transmission electron microscope Zeiss EM 900 24 h after: calcium electroporation (0.5 mM, 1000 V/cm); calcium alone (0.5 mM); electroporation alone (1000 V/cm); untreated cells. Cells were electroporated in suspension. Cells were fixed in 2.5% glutaraldehyde for 12 h, washed in 0.1 M phosphate buffer (pH 7.4) 3 times, post-fixed in 1% osmium tetroxide for 30 min and dehydrated through a graded series of acetone. Samples were embedded in Epon (EMbed 812 Kit, Electron Microscopy Sciences).

### Calcium electroporation *in vivo*

Experiments were conducted in accordance with European Convention for the Protection of Vertebrate Animals used for Experimentation and with permission from the Danish Animal Experiments Inspectorate. RD cells (5 × 10^6^ in 100 μl PBS) were injected subcutaneously in the flank of 8- to 12-week-old NMRI-*Foxn1^nu^* mice (Harlan and bred at Department of Oncology, Herlev Hospital, Denmark). Hypnorm-dormicum (VetaPharma and Roche) was used as anesthesia. Tumor size was measured using a Vernier Caliper and calculated according to the equation: ab^2^π/6 (a, largest diameter; b, largest diameter perpendicular to a). Mice were randomized in four groups when the tumors were above 85 mm^3^: (1) calcium chloride (168 mmol/L) injection; (2) calcium chloride injection (168 mmol/L) followed by electroporation (8 pulses of 100 μs at 1000 V/cm, and 1 Hz) using a 6 mm plate electrode and a square wave electroporator (Cliniporator, IGEA); (3) electroporation alone (parameters as above); (4) untreated. Injection volume of CaCl_2_ was equivalent to 50% of the tumor volume. Response of the treatment was followed by measuring tumor size twice a week.

### Statistics

Statistical analyses were performed using SPSS software (version 19). Differences in viability *in vitro*, in intracellular calcium level *in vitro*, and in florescent signal intensity were assessed by 2-way analysis of variance (ANOVA) with post least-squares-means test with Bonferroni correction. In the event of violation of the assumption of data being normally distributed, data was log transformed. Differences in tumor volume after treatment *in vivo* were evaluated with linear mixed model.

## SUPPLEMENTARY MATERIALS FIGURES AND TABLES



## Abbreviations

EP: electroporation
CaEP: calcium electroporation
NCX: sodium/calcium exchanger
PMCA: plasma membrane Ca^2+^-ATPase
RyR: ryanodine receptor
SERCA: sarco/endoplasmic reticulum Ca^2+^-ATPase
CLSM: Confocal laser scanning microscopy
TEM: transmission electron microscope
PTP: permeability transition pores
rER: rough endoplasmic reticulum

## References

[1] Weiss SW, Goldblum J, Weiss SW, Goldblum JR (2001). Rhabdomyosarcoma. Enzinger and Weiss’s soft tissue tumors.

[2] Wexler LH, Crist WM, Helman LJ, Pizzo PA, Poplack DG (2002). Rhabdomyosarcoma and the undifferentiated sarcomas. Principles and practice of pediatric oncology.

[3] Casas M, Figueroa R, Jorquera G, Escobar M, Molgo J, Jaimovich E (2010). IP3-dependent, post-tetanic calcium transients induced by electrostimulation of adult skeletal muscle fibers. J Gen Physiol.

[4] Clapham DE (2007). Calcium signaling. Cell.

[5] Strehler EE, Treiman M (2004). Calcium pumps of plasma membrane and cell interior. Curr Mol Med.

[6] Görlach A, Bertram K, Hudecova S, Krizanova O (2015). Calcium and ROS: A mutual interplay. Redox Biol.

[7] Frandsen SK, Gissel H, Hojman P, Tramm T, Eriksen J, Gehl J (2012). Direct therapeutic applications of calcium electroporation to effectively induce tumor necrosis. Cancer Res.

[8] Zielichowska A, Daczewska M, Saczko J, Michel O, Kulbacka J (2016). Applications of calcium electroporation to effective apoptosis induction in fibrosarcoma cells and stimulation of normal muscle cells. Bioelectrochemistry.

[9] Zhivotovsky B, Orrenius S (2011). Calcium and cell death mechanisms: a perspective from the cell death community. Cell Calcium.

[10] Frandsen SK, Krüger MA, Mangalanathan UM, Tramm T, Mahmood F, Novak I (2017). Normal and Malignant Cells Exhibit Differential Responses to Calcium Electroporation. Cancer Res.

[11] Hansen EL, Sozer EB, Romeo S, Frandsen SK, Vernier PT, Gehl J (2015). Dose-dependent ATP depletion and cancer cell death following calcium electroporation, relative effect of calcium concentration and electric field strength. PLoS One.

[12] Frandsen SK, Gibot L, Madi M, Gehl J, Rols MP (2015). Calcium Electroporation: Evidence for Differential Effects in Normal and Malignant Cell Lines, Evaluated in a 3D Spheroid Model. PLoS One.

[13] Frandsen SK, Gehl J (2017). Effect of calcium electroporation in combination with metformin *in vivo* and correlation between viability and intracellular ATP level after calcium electroporation *in vitro*. PLoS One.

[14] Guerini D (1998). The significance of the isoforms of plasma membrane calcium ATPase. Cell Tissue Res.

[15] Gao Y, Wheatly MG (2004). Characterization and expression of plasma membrane Ca2+ ATPase (PMCA3) in the crayfish Procambarus clarkii antennal gland during molting. J Exp Biol.

[16] Greeb J, Shull GE (1989). Molecular cloning of a third isoform of the calmodulin-sensitive plasma membrane Ca2+–transporting ATPase that is expressed predominantly in brain and skeletal muscle. J Biol Chem.

[17] Roberts-Thomson SJ, Curry MC, Monteith GR (2010). Plasma membrane calcium pumps and their emerging roles in cancer. World J Biol Chem.

[18] Brini M, Carafoli E (2011). The Plasma Membrane Ca2+ ATPase and the Plasma Membrane Sodium Calcium Exchanger Cooperate in the Regulation of Cell Calcium. Cold Spring Harb Perspect Biol.

[19] Eymery A, Callanan M, Vourc’h C (2009). The secret message of heterochromatin: new insights into the mechanisms and function of centromeric and pericentric repeat sequence transcription. Int J Dev Biol.

[20] Kroemer G (2006). Mitochondria in cancer. Oncogene.

[21] Guerini D, Garcia-Martin E, Zecca A, Guidi F, Carafoli E (1998). The calcium pump of the plasma membrane: membrane targeting, calcium binding sites, tissue-specific isoform expression. Acta Physiol ScandSuppl.

[22] Meng X, Riordan NH, Riordan HD, Mikirova N, Jackson J, González MJ, Miranda-Massari JR, Mora E, Trinidad Castillo W (2004). Cell membrane fatty acid composition differs between normal and malignant cell lines. P R Health Sci J.

[23] Gehlert S, Bloch W, Suhr F (2015). Ca2+– dependent regulations and signaling in skeletal muscle: from electro-mechanical coupling to adaptation. Int J Mol Sci.

[24] Monteith GR, Davis FM, Roberts-Thomson SJ (2012). Calcium Channels and Pumps in Cancer: Changes and Consequences. J Biol Chem.

[25] Rohwedel J, Maltsev V, Bober E, Arnold HH, Hescheler J, Wobus AM (1994). Muscle cell differentiation of embryonic stem cells reflects myogenesis *in vivo*: developmentally regulated expression of myogenic determination genes and functional expression of ionic currents. Dev Biol.

[26] Calì T, Ottolini D, Brini M, Chakraborti S, Dhalla NS (2016). The Plasma Membrane Ca2+ ATPases: Isoform Specificity and Functional Versatility. Regulation of Ca2+– ATPases,V-ATPases and F-ATPases in Advances in Biochemistry in Health and Disease.

[27] Aung CS, Kruger WA, Poronnik P, Roberts-Thomson SJ, Monteith GR (2007). Plasma membrane Ca2+– ATPase expression during colon cancer cell line differentiation. Biochem Biophys Res Commun.

[28] Ribiczey P, Tordai A, Andrikovics H, Filoteo AG, Penniston JT, Enouf J, Enyedi A, Papp B, Kovács T (2007). Isoform-specific up-regulation of plasma membrane Ca2+ ATPase expression during colon and gastric cancer cell differentiation. Cell Calcium.

[29] Cifuentes F, Vergara J, Hidalgo C (2000). Sodium/calcium exchange in amphibian skeletal muscle fibers and isolated transverse tubules. Am J Physiol Cell Physiol.

[30] Wen J, Pang Y, Zhou T, Qi X, Zhao M, Xuan B, Meng X, Guo Y, Liu Q, Liang H, Li Y, Dong H, Wang Y (2016). Essential role of Na+/Ca2+ exchanger 1 in smoking-induced growth and migration of esophageal squamous cell carcinoma. Oncotarget.

[31] Dong H, Shim KN, Li JM, Estrema C, Ornelas TA, Nguyen F (2010). Molecular mechanisms underlying Ca2+– mediated motility of human pancreatic duct cells. Am J Physiol Cell Physiol.

[32] Iwamoto T, Watanabe Y, Kita S, Blaustein MP (2007). Na+/Ca2+ exchange inhibitors: a new class of calcium regulators. Cardiovasc Hematol Disord Drug Targets.

[33] Abdul M, Ramlal S, Hoosein N (2008). Ryanodine receptor expression correlates with tumor grade in breast cancer. Pathol Oncol Res.

[34] Mariot P, Prevarskaya N, Roudbaraki MM, Le Bourhis X, Van Coppenolle F, Vanoverberghe K, Skryma R (2000). Evidence of functional ryanodine receptor involved in apoptosis of prostate cancer (LNCaP) cells. Prostate.

[35] Drees BE, Andrews KM, Beckerlea MC (1999). Molecular Dissection of Zyxin Function Reveals Its Involvement in Cell Motility. J Cell Biol.

[36] Yoshigi M, Hoffman LM, Jensen CC, Yost HJ, Beckerle MC (2005). Mechanical force mobilizes zyxin from focal adhesions to actin filaments and regulates cytoskeletal reinforcement. J Cell Biol.

[37] Hirota T, Morisaki T, Nishiyama Y, Marumoto T, Tada K, Hara T, Masuko N, Inagaki M, Hatakeyama K, Saya H (2000). Zyxin, a Regulator of Actin Filament Assembly, Targets the Mitotic Apparatus by Interacting with H-Warts/Lats1 Tumor Suppressor. J Cell Biol.

[38] Crawford AW, Beckerle MC (1991). Purification and characterization of zyxin, an 82,000-Dalton component of adherens junctions. J Biol Chem.

[39] Smith MA, Blankman E, Gardel ML, Luettjohann L, Waterman CM, Beckerle MC (2010). A zyxin-mediated mechanism for actin stress fiber maintenance and repair. Dev Cell.

[40] Oldenburg J, van der Krogt J, Twiss F, Bongaarts A, Habani Y, Slotman JA (2015). VASP, zyxin and TES are tension-dependent members of Focal Adherens Junctions independent of the α-catenin-vinculin module. Sci Rep.

[41] Yonemura S (2017). Actin filament association at adherens junctions. J Med Invest.

[42] Ghosh S, Kollar B, Nahar T, Suresh Babu S, Wojtowicz A, Sticht C, Gretz N, Wagner AH, Korff T, Hecker M (2015). Loss of the mechanotransducer zyxin promotes a synthetic phenotype of vascular smooth muscle cells. J Am Heart Assoc.

[43] Indran IR, Tufo G, Pervaiz P, Brenner C (2011). Recent advances in apoptosis, mitochondria and drug resistance in cancer cells. Biochim Biophys Acta.

[44] Chiche J, Rouleau M, Gounon P, Brahimi-Horn MC, Pouysségur J, Mazure NM (2010). Hypoxic enlarged mitochondria protect cancer cells from apoptotic stimuli. J Cell Physiol.

[45] Gogvadze V, Orrenius S, Zhivotovsky B (2009). Mitochondria as targets for cancer chemotherapy. Semin Cancer Biol.

[46] Weinberg SE, Chandel NS (2015). Targeting mitochondria metabolism for cancer therapy. Nat Chem Biol.

[47] Gerace L, Gilmore R, Johnson A, Lazarow P, Neupert W, O’shea E, Alberts B, Johnson A, Lewis J, Raff M, Roberts K, Walter P (2002). Intracellular compartments and protein sorting. Molecular Biology of the Cell.

[48] Mrschtik M, Ryan KM (2015). Lysosomal proteins in cell death and autophagy. FEBS J.

[49] Luzio JP, Pryor PR, Bright NA (2007). Lysosomes: fusion and function. Nat Rev Mol Cell Biol.

[50] Parzych KR, Klionsky DJ (2014). An overview of autophagy: morphology, mechanism, and regulation. Antioxid Redox Signal.

[51] Falk H, Forde PF, Bay ML, Mangalanathan UM, Hojman P, Soden DM, Gehl J (2017). Calcium electroporation induces tumor eradication, long-lasting immunity and cytokine responses in the CT26 colon cancer mouse model. Oncoimmunology.

[52] Falk H, Matthiessen LW, Wooler G, Gehl J (2017). Calcium electroporation for treatment of cutaneous metastases; a randomized double-blinded phase II study, comparing the effect of calcium electroporation with electrochemotherapy. Acta Oncol.

[53] Kulbacka J, Paczuska J, Rembiałkowska N, Saczko J, Kiełbowicz Z, Kinda W (2017). Electrochemotherapy combined with standard and CO2 laser surgeries in canine oral melanoma. Slov Vet Res.

[54] Bouché M, Senni MI, Grossi AM, Zappelli F, Polimeni M, Arnold HH, Cossu G, Molinaro M (1993). TPA-induced differentiation of human rhabdomyosarcoma cells: expression of the myogenic regulatory factors. Exp Cell Res.

[55] Elkalaf M, Anděl M, Trnka J (2013). Low glucose but not galactose enhances oxidative mitochondrial metabolism in C2C12 myoblasts and myotubes. PLoS One.

