# Correction: Calcium electroporation for treatment of sarcoma in preclinical studies

**DOI:** 10.18632/oncotarget.27158

**Published:** 2019-08-20

**Authors:** Anna Szewczyk, Julie Gehl, Malgorzata Daczewska, Jolanta Saczko, Stine Krog Frandsen, Julita Kulbacka

**Affiliations:** ^1^ Department of Animal Developmental Biology, Institute of Experimental Biology, University of Wroclaw, Wroclaw, Poland; ^2^ Center for Experimental Drug and Gene Electrotransfer (C*EDGE), Department of Clinical Oncology and Palliative Care, Zealand University Hospital, Roskilde, Denmark; ^3^ Department of Clinical Medicine, Faculty of Health and Medical Sciences, University of Copenhagen, Copenhagen, Denmark; ^4^ Department of Oncology, Herlev and Gentofte Hospital, University of Copenhagen, Herlev, Denmark; ^5^ Department of Medical Biochemistry, Wroclaw Medical University, Wroclaw, Poland

**This article has been corrected:** In the Abstract section, the 2nd sentence of the 3rd paragraph has been corrected to include the following: “PMCA expression was lower in untreated malignant cells than normal cells. CaEP caused decreased expression of NCX1 in malignant cells and RyR1 in both cell lines whereas normal cells exhibited increased expression of NCX1 after CaEP.”

## ABSTRACT

Calcium electroporation (CaEP) describes the use of electric pulses (electroporation) to transiently permeabilize cells to allow supraphysiological doses of calcium to enter the cytosol. Calcium electroporation has successfully been investigated for treatment of cutaneous metastases in a clinical study. This preclinical study explores the possible use of calcium electroporation for treatment of sarcoma.

A normal murine muscle cell line (C2C12), and a human rhabdomyosarcoma cell line (RD) were used in the undifferentiated and differentiated state. Electroporation was performed using 8 pulses of 100 µs at 600–1000 V/cm; with calcium (0, 0.5, 1, and 5 mM). Viability was examined by MTS assay, intracellular calcium levels were measured, and expression of plasma membrane calcium ATPase (PMCA) was investigated using western blotting. Calcium/sodium exchanger (NCX1), ryanodine receptor (RyR1) expression and cytoskeleton structure (zyxin/actin) were assessed by immunofluorescence. CaEP efficiency on RD tumors was tested *in vivo* in immuno-deficient mice.

CaEP was significantly more efficient in RD than in normal cells. PMCA expression was lower in untreated malignant cells than normal cells. CaEP caused decreased expression of NCX1 in malignant cells and RyR1 in both cell lines whereas normal cells exhibited increased expression of NCX1 after CaEP. Calcium electroporation also affected cytoskeleton structure in malignant cells.

This study showed that calcium electroporation is tolerated significantly better in normal musclreatment this could potentially be used in connection with sare cells than sarcoma cells and as an inexpensive and simple cancer tcoma surgery for local treatment.

Original article: Oncotarget. 2018; 9:11604–11618. 11604-11618 . https://doi.org/10.18632/oncotarget.24352

