# Supplementary material for: Calcium electroporation for treatment of sarcoma in preclinical studies
Source: Oncotarget. 2018 Jan 30;9(14):11604–18. doi: 10.18632/oncotarget.24352 (PMC5837766; doi:10.18632/oncotarget.24352)
Supplement: Supplementary file 1 [file oncotarget-09-11604-s001.pdf]

Calcium electroporation for treatment of sarcoma in preclinical studies

SUPPLEMENTARY MATERIALS

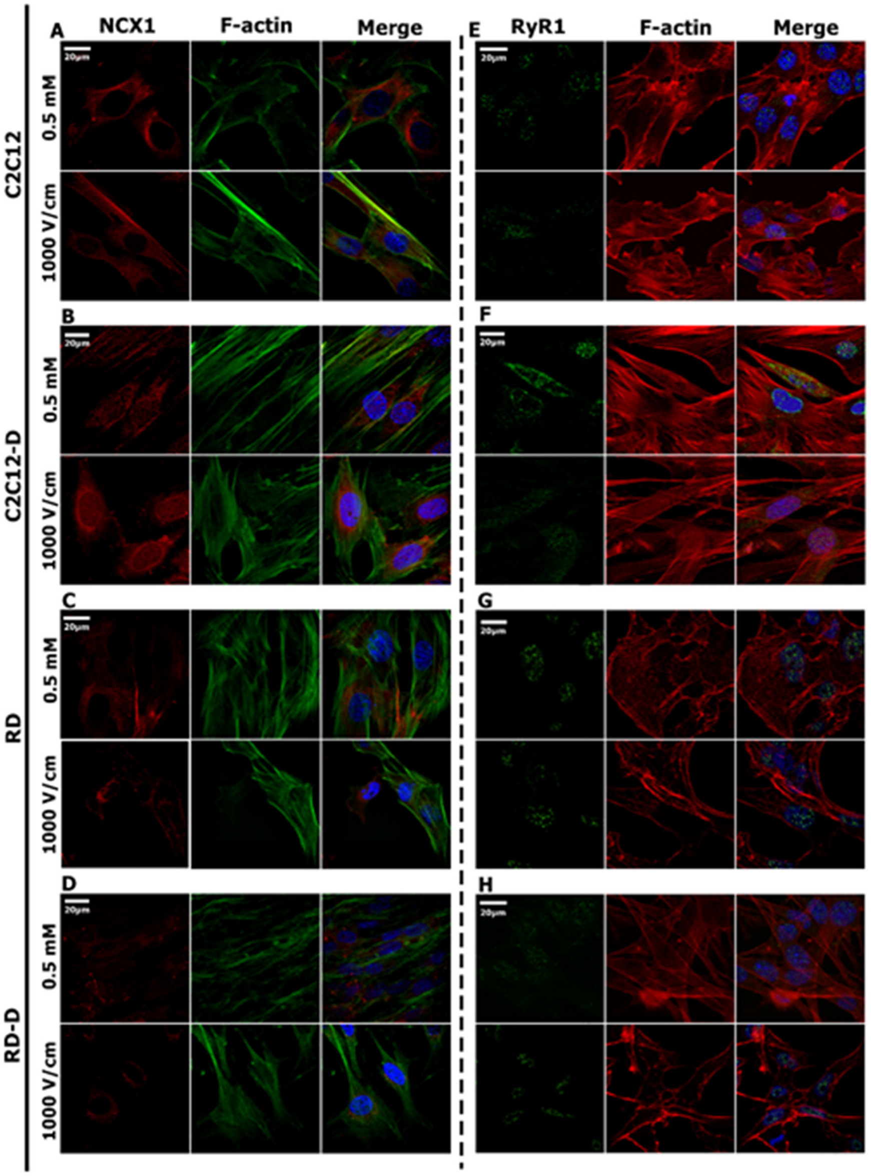

Supplementary Figure 1: Immunofluorescent visualization of calcium/sodium exchanger (NCX1) and ryanodine receptor (Ryr1) in normal (C2C12, C2C12-D) and malignant (RD, RD-D) cells after calcium incubation alone and electroporation alone. The left panel: CLSM images present changes in NCX1 (red) intracellular localization and signal intensity 24 h after calcium incubation (0.5 mM) and electroporation (1000 V/cm) for cell lines: C2C12 (**A**), C2C12-D (**B**), RD (**C**), RD-D (**D**). The right panel: CLSM images present changes in RyR1 (green) intracellular localization and signal intensity 24 h after calcium incubation (0.5 mM) and electroporation (1000 V/cm) for cell lines: C2C12 (**E**), C2C12-D (**F**), RD (**G**), RD-D (**H**). Both channels are colocalized with F-actin fibers (green-left panel, red-right panel); 20 μm; *n* = 3–5. The signal intensity was analyzed by ImageJ software (shown in Figure 4).

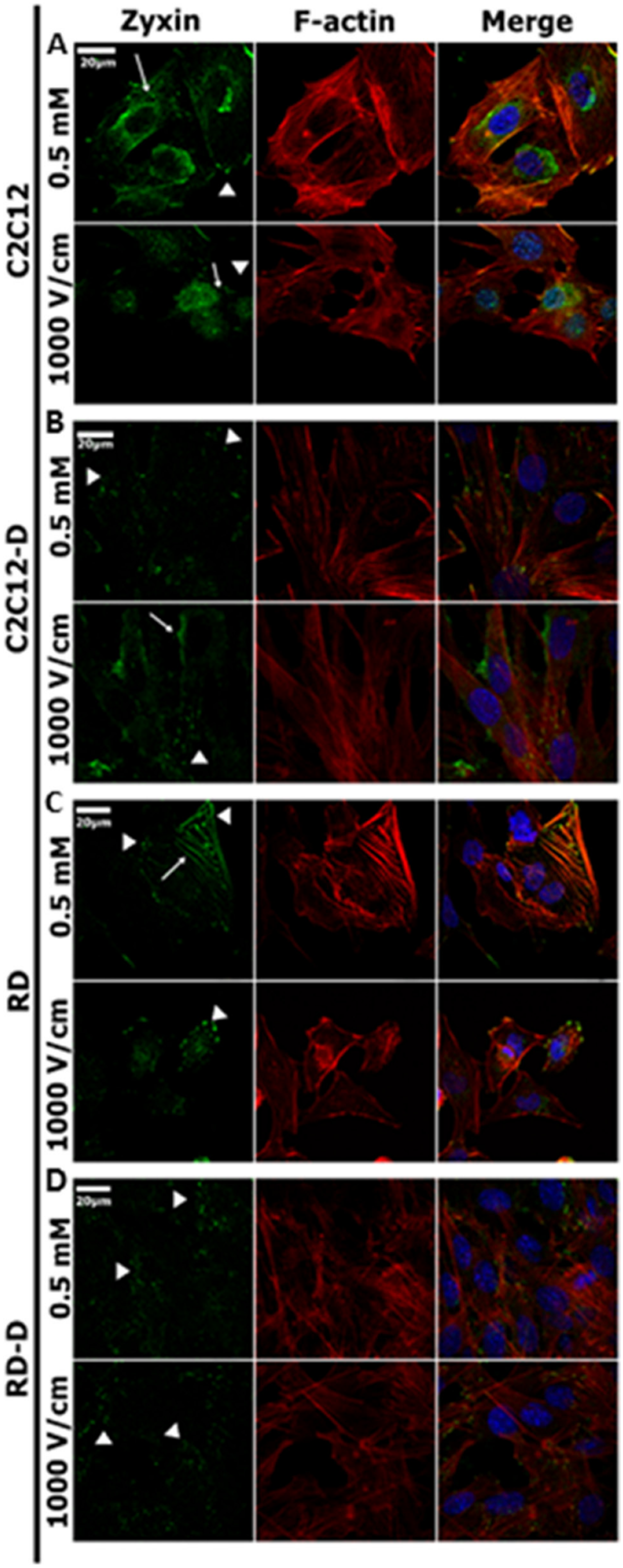

Supplementary Figure 2: CLSM visualization of the rearrangement of zyxin and F-actin structure in normal (C2C12, C2C12) and malignant (RD, RD-D) cells. Immunofluorescence evaluation of zyxin (green) and actin (red) fibers expression for undifferentiated cell lines: C2C12 (A), RD (C) and differentiated cell lines: C2C12-D (B), RD-D (D) 24 h after calcium incubation alone (0.5 mM) and electroporation alone application (1000 V/cm). The white short arrows indicate focal adhesions, white long arrows indicate actin stress fibers; 20 µm; *n* = 3–5. The signal intensity was analyzed by ImageJ software (shown in Figure 5).
